# Seasonal prediction of the distribution of three major malaria vectors in China: Based on an ecological niche model

**DOI:** 10.1371/journal.pntd.0011884

**Published:** 2024-01-18

**Authors:** Qi An, Yuepeng Li, Zhuo Sun, Xiang Gao, Hongbin Wang

**Affiliations:** 1 College of Veterinary Medicine, Northeast Agricultural University, Harbin, People’s Republic of China; 2 Key Laboratory of the Provincial Education Department of Heilongjiang for Common Animal Disease Prevention and Treatment, College of Veterinary Medicine, Northeast Agricultural University, Harbin, People’s Republic of China; The University of Hong Kong, CHINA

## Abstract

Against the backdrop of a global malaria epidemic that remains severe, China has eradicated indigenous malaria but still has to be alert to the risk of external importation. Understanding the distribution of vectors can provide an adequate and reliable basis for the development and implementation of vector control strategies. However, with the decline of malaria prevalence in recent years, the capacity of vector monitoring and identification has been greatly weakened. Here we have used new sampling records, climatic data, and topographic data to establish ecological niche models of the three main malaria vectors in China. The model results accurately identified the current habitat suitability areas for the three species of *Anopheles* and revealed that in addition to precipitation and temperature as important variables affecting the distribution of *Anopheles* mosquitoes, topographic variables also influenced the distribution of *Anopheles* mosquitoes. *Anopheles sinensis* is the most widespread malaria vector in China, with a wide region from the northeast (Heilongjiang Province) to the southwest (Yunnan Province) suitable for its survival. Suitable habitat areas for *Anopheles lesteri* are concentrated in the central, eastern, and southern regions of China. The suitable habitat areas of *Anopheles minimus* are the smallest and are only distributed in the border provinces of southern China. On this basis, we further assessed the seasonal variation in habitat suitability areas for these three major malaria vectors in China. The results of this study provide new and more detailed evidence for vector monitoring. In this new era of imported malaria prevention in China, regular reassessment of the risk of vector transmission is recommended.

## Author summary

Malaria, as one of the most important insect-borne infectious diseases in tropical and subtropical regions, poses a serious disease burden worldwide. Malaria has been prevalent in China for more than 4,000 years, seriously endangering the health of the Chinese people and restricting socio-economic development. Nowadays, there are three major species of malaria transmission in China, *Anopheles sinensis*, *Anopheles lesteri*, and *Anopheles minimus*. Due to vector control and climate change in recent years, the distribution range of these *Anopheles* mosquitoes has changed. Here, we applied an ecological niche model to simulate the potential distribution as well as seasonal variation of three species of *Anopheles* and to identify the factors affecting their survival. The results revealed that precipitation, temperature, and topographic variables were important influences on the distribution of *Anopheles* mosquitoes. A wide region from the northeast (Heilongjiang Province) to the southwest (Yunnan Province) is suitable for *An*. *sinensis*. Suitable habitat areas for *An*. *lesteri* are concentrated in the central, eastern, and southern regions of China. The suitable habitat areas of *An*. *minimus* are the smallest, which are only distributed in the border provinces of southern China. This study provides new information for policymakers to develop vector control measures.

## Introduction

Malaria is a life-threatening disease caused by *Plasmodium* parasites and is transmitted worldwide through the female *Anopheles* mosquitoes [1]. The initial symptoms of malaria usually appear 10–15 days after being bitten by infected mosquitoes. Non-specific symptoms such as fever, headache, nausea, and muscle soreness may occur in patients infected with *Plasmodium*, which also makes it difficult to diagnose malaria at the initial stage [2]. If the initial infection of an individual is not effectively controlled, it will progress to a severe and complicated form of malaria, causing respiratory distress, jaundice, severe anemia, and renal failure, which can lead to death [3]. Five species of *Plasmodium* cause malaria in humans, with *Plasmodium falciparum* and *Plasmodium vivax* being the most harmful. In the world, *P*. *falciparum* is the most lethal malaria parasite, and *P*. *vivax* is the most widespread malaria parasite. The latest World Malaria Report has estimated that there were 247 million malaria cases and 619,000 malaria deaths globally in 2021, with the majority concentrated in the African region [4]. Despite the severe global disease burden caused by malaria, effective vector control and the use of prophylactic antimalarial drugs have made significant progress in reducing morbidity and mortality from this disease over the past two decades [5]. The number of countries reporting less than 1,000 indigenous cases was only 13 in 2000, compared to 33 in 2020 and 35 in 2021 [4,5].

Malaria is one of the most serious infectious diseases in the history of China. It was once widely prevalent in China, especially in rural areas [6]. The earliest cases of malaria can be traced back to about 4,000 years ago [7]. In the 1940s, the malaria epidemic in China was so severe that more than 30 million people were infected with the disease each year [8]. In the decades since the founding of New China, the Chinese government has made tremendous efforts to control malaria. As a result of the implementation of a comprehensive strategy for malaria control, as well as socio-economic changes in China and changes in the natural environment affecting malaria transmission, the epidemic has been effectively controlled, with a gradual decline in incidence and a gradual shrinkage of endemic areas [9–11]. In 2010, in view of the prevailing malaria epidemic trends and in response to the initiative of the High-Level Meeting on the Millennium Development Goals (MDGs) to eradicate malaria globally, the Chinese government formulated the National Malaria Elimination Action Plan (2010–2020) (NMEAP), intending to implement comprehensive malaria elimination efforts and achieve the goal of malaria elimination nationwide by 2020 [12]. In the years that followed, the indigenous malaria epidemic in China was dramatically reduced, with zero indigenous cases achieved for the first time in 2017 [13]. On 30 June 2021, the World Health Organization announced that China had been certified as malaria-free. China is the first country in the Western Pacific region of the World Health Organization to obtain malaria-free certification in the past 30 years, which is undoubtedly a major milestone in the history of malaria elimination in the world and public health in China [14].

Malaria-free certification is not the ultimate goal of public health in China, but a new starting point for the prevention of imported malaria cases. After malaria elimination, imported malaria is still widely distributed in China. Malaria cases were reported from all 31 provinces in 2021 [15], and 26 provinces in 2022 [16], mainly in the Guangdong Province, Yunnan Province, Sichuan Province, Zhejiang Province, Henan Province, and Shanghai Municipality. From 2017 to 2022, the number of imported malaria in China was 2858, 2671, 2673, 1085, 798, and 844, respectively [15–20]. In general, the number of imported cases has shown a downward trend in recent years, but this may be attributed to the global COVID-19 outbreak. At present, the prevention and control of COVID-19 in China have become normalized, and international exchanges have gradually resumed. Until malaria is eradicated globally, China still needs to be alert to the risk of imported malaria, where introduced *Plasmodium* may lead to the re-emergence of indigenous malaria, which has already occurred in countries where malaria has been eliminated [21]. If the malaria transmission vector in China still exists, the risk of malaria import and retransmission will exist for a long time. A reasonable vector control strategy should continue to be developed and implemented as it is highly effective in preventing infection and reducing the spread of disease. In order to implement appropriate and targeted vector control strategies, a better understanding of the distribution and biology of malaria vectors is necessary.

In the long struggle against malaria, China has improved its malaria vector monitoring system, which consists of national and provincial monitoring sites [22]. The malaria vector monitoring program includes the *Anopheles* mosquito population and density monitoring [23]. *Anopheles* mosquito population monitoring adopts the method of overnight mosquito trapping utilizing mosquito trapping lamps and is carried out once a year in different eco-geographical environments during the peak mosquito season. *Anopheles* mosquito density monitoring adopts the overnight human trapping method, which is carried out during the peak mosquito season every year, once every half a month, one night each time. After decades of climate and land change and malaria control, traces of some *Anopheles* mosquitoes have gradually disappeared [24]. At present, only four species of *Anopheles* are considered to be the main malaria vectors in China, in descending order of distribution: *Anopheles sinensis*, *Anopheles lesteri*, *Anopheles minimus*, and *Anopheles dirus* [5]. *An*. *sinensis* is an outdoor resting and biting species, which mostly inhabits rice planting areas, and can be recorded between northeast to Liaoning Province and southwest to Yunnan Province [25]. Studies have indicated that the habitat suitability areas of *An*. *sinensis* tend to expand gradually with climate change [26,27]. *An*. *lesteri* is the main malaria vector at low altitudes in the 22°N to 33°N range [28,29]. In recent years, the distribution areas of *An*. *lesteri* have been gradually reduced due to climatic and environmental changes and human activities [30]. *An*. *minimus* is active in southern China below 25°N and mainly inhabits marshes, ponds, and streams in jungle areas [31]. *An*. *dirus* has the smallest distribution range and is only found in Hainan Province [29].

Vector control has played an invaluable role in the malaria elimination phase. Long-term vector monitoring capacity is still needed during periods of no indigenous malaria, thus reducing the risk of re-transmission of imported malaria. Understanding the distribution of vectors can provide an adequate and reliable basis for the development and implementation of vector control strategies. However, with the decline of malaria prevalence in recent years, the capacity of vector monitoring and identification has been greatly weakened [29]. The application of ecological niche models to predict the distribution of malaria vectors can provide a powerful complement to vector monitoring efforts. In addition, analyzing the seasonal impact of climate on malaria vector distribution will optimize the use of vector control resources, allowing vector control strategies to be implemented with precision and efficiency to address the risk of external malaria transmission. In this study, we applied an ecological niche model to predict the distribution and seasonal variation of three major malaria vectors in China, including *An*. *sinensis*, *An*. *lesteri*, and *An*. *minimus*. The distribution points of *An*. *dirus* were too few to be included in this study.

## Materials and methods

### Occurrence data collection

A comprehensive and systematic literature search was conducted on CNKI, Web of Science, and Google Scholar using the keywords “malaria vector” AND “China”, “*Anopheles lesteri*” AND “China”, “*Anopheles minimus*” AND “China”, and “*Anopheles sinensis*” AND “China”, for the period 2001 to 2021. The search results were examined individually. Literature on field surveys of *Anopheles* mosquitoes (containing GPS coordinates of sampling sites or locations of sampling sites at the county level and below) was included in the database. Literature containing records of *Anopheles* mosquitoes from laboratory cultivation and museum collection and literature with unspecified field sampling sites were excluded. For sampled points with specific GPS coordinates, the coordinates were converted to decimal form and extracted. If only the name of the sampling site is included, use Google Maps to get the decimal coordinates of the site. The occurrence data of each species of *Anopheles* were cleaned and sorted and finally summarized into a CSV format file.

The “Trim duplicate occurrences” function in the ENMTools software was used to eliminate duplicate data and reduce spatial autocorrelation [32], ensuring that only one occurrence record in each grid. Finally, a total of 95 occurrence records of *An*. *lesteri*, 148 occurrence records of *An*. *minimus*, and 446 occurrence records of *An*. *sinensis* was used to construct the subsequent ecological niche model (S1 Table).

### Model variables collection

Climate and topographic variables affect the habitat conditions of mosquitoes and have been used to predict their habitat range by ecological niche models [33–35]. The monthly mean temperature dataset, monthly precipitation dataset, and land cover fraction dataset are sourced from the National Tibetan Plateau Data Centre (https://data.tpdc.ac.cn/home) [36–38]. The monthly mean temperature dataset and monthly precipitation dataset are both monthly data for China, from 1901.1 to 2021.12. To be consistent with the time range of the occurrence data, the average values of 252 months from 2001 to 2021 are calculated for the monthly average temperature and monthly precipitation. The land cover fraction dataset provides eight types of land cover in China, including Forest, Grassland and Shrub, Croland, Wetland, Water, Construction, Bare land, Permanent Snow and Ice, with a time series of 2001–2018. Annual averages were calculated for each land cover type for subsequent modeling. Elevation data were obtained from SRTM, downloaded from the WorldClim website (https://worldclim.org/), and cropped from worldwide to China-wide using the mask extraction tool of ArcGIS 10.2. The resolution for all variables used for modeling is 30 arc seconds, approximately 1km (Table 1).

**Table 1:** Variables used in the model.

Code	Variable Name	Source
bare_land	Bare Land	National Tibetan Plateau Data Center
construction	Construction	National Tibetan Plateau Data Center
cropland	Cropland	National Tibetan Plateau Data Center
elev	Elevation	WorldClim version 2.1
forest	Forest	National Tibetan Plateau Data Center
gs	Grassland and Shrub	National Tibetan Plateau Data Center
pre	Monthly Precipitation	National Tibetan Plateau Data Center
psi	Permanent Snow and Ice	National Tibetan Plateau Data Center
tmp	Monthly Mean Temperature	National Tibetan Plateau Data Center
water	Water	National Tibetan Plateau Data Center
wetland	Wetland	National Tibetan Plateau Data Center

### Ecological niche modeling

Maxent, one of the most widely used models in ecology, has excellent predictive performance and can produce distribution maps of comparable accuracy to ensemble methods [39]. Maxent performs well in estimating occupancy probabilities and even outperforms the other methods on small sample sizes [40]. In addition, maxent is capable of modeling complex nonlinear relationships between response variables and predictions, and the probability distributions in the model have concise mathematical definitions that are easy to analyze [41]. MaxEnt 3.4.4 was employed to predict the potential distribution of *An*. *lesteri*, *An*. *minimus*, and *An*. *sinensis*. The model was set up as follows: the occurrence data of each species of *Anopheles* were split into training data and test data on a scale of 80–20. The regularization multiplier was adjusted to 1, features were automatically selected by the model program and the output format was logistic. The model of each species of *Anopheles* was repeated 10 times, with the replicated run type being Bootstrap, and the average of the 10 results was taken as the final prediction. Model performance was evaluated by area under the receiver operating characteristic curve (AUC). The range of AUC value is 0 to 1, 1 represents the extreme case of perfect accuracy of the model, 0.9~1.0 indicates good model fitting, 0.7~0.8 indicates general model fitting, 0.6~0.7 indicates poor model fitting, and 0.5~0.6 indicates model fitting failure [42]. The percent contribution and the permutation importance give estimates of the relative contributions of the environmental variables to the maxent model. The response curves show how the predicted probability of presence changes as each environmental variable is varied. Habitat suitability represents an index of the suitability of the area for the species, which reveals the probability that the species be present in the area. habitat suitability values range from 0–1, with values closer to 1 indicating that the area is more suitable for the species and closer to 0 indicating that the area is less suitable for the species.

### Prediction of seasonal variation in habitat suitability maps for *Anopheles* mosquitoes

The survival of mosquitoes is mainly controlled by temperature and precipitation, so we predict the seasonal variation trend of the distribution range of *Anopheles* mosquitoes by the fluctuation of monthly mean temperature and monthly precipitation. Data from the monthly mean temperature dataset and the monthly precipitation dataset were intercepted for the range 2001 to 2021 to calculate the average of monthly mean temperature and monthly precipitation for the 21 years from January to December. The model output was converted to raster files in ArcGIS 10.2 to produce habitat suitability maps for each species of *Anopheles*. Based on the response curves and equal training sensitivity and specificity logistic thresholds calculated from the model iterations, suitable ranges of monthly mean temperature and monthly precipitation were determined for each species of *Anopheles*. Using the raster calculator, raster maps of the suitable monthly mean temperature and suitable monthly precipitation for each species of *Anopheles* from January to December were obtained. The habitat suitability maps were overlaid with these two raster maps and the intersection was taken to produce a monthly habitat suitability map for each species of *Anopheles*.

## Result

### Map of the distribution points of the three malaria vectors in China

**Fig 1:**
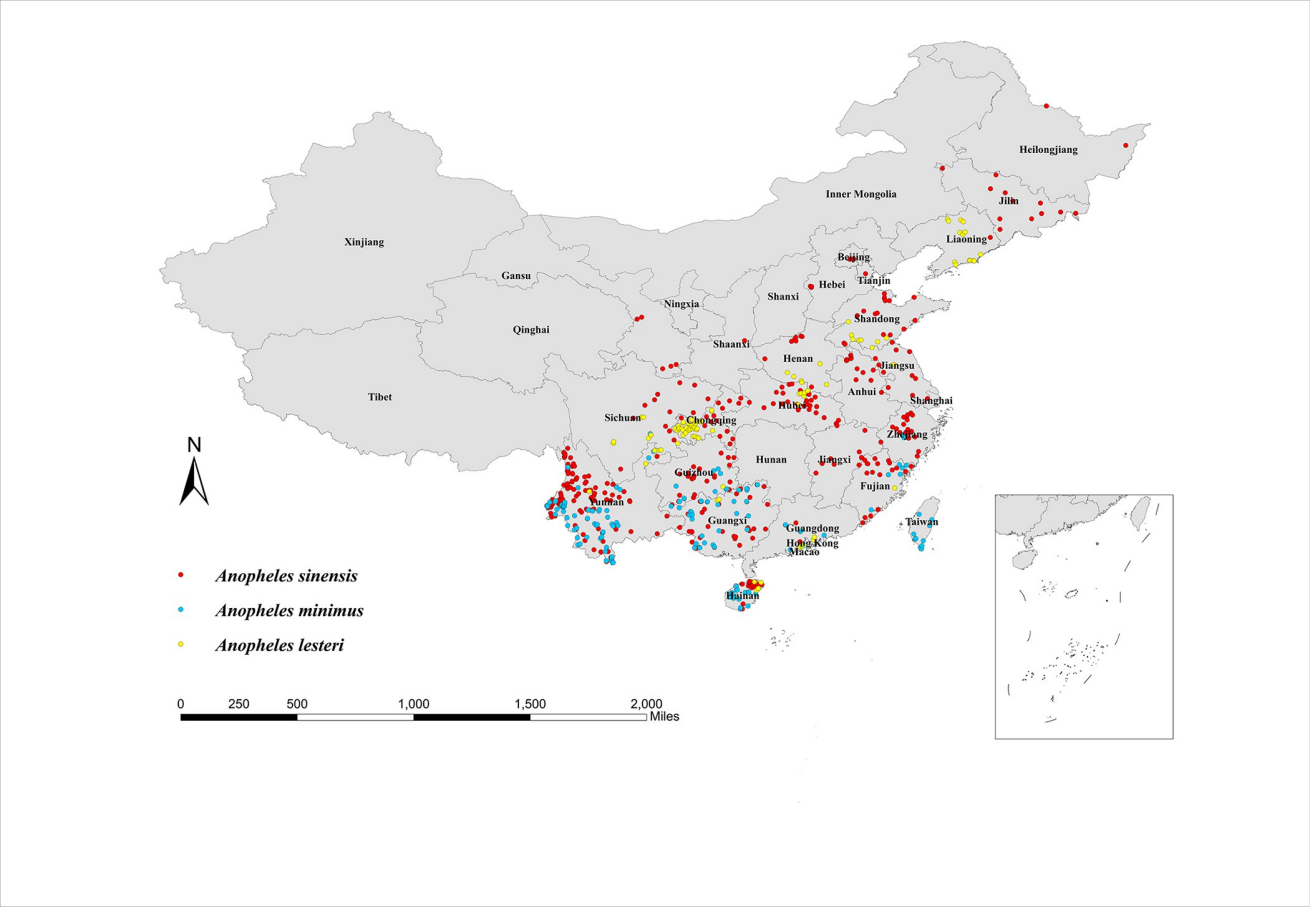
Map of the distribution points of the three malaria vectors in China. The red points depict *Anopheles sinensis*, the blue points depict *Anopheles minimus*, and the yellow points depict *Anopheles lesteri*. The base layer of the map is sourced from the National Catalogue Service For Geographic Information of the Ministry of Natural Resources of the People’s Republic of China (https://www.webmap.cn/mapDataAction.do?method=forw&resType=5&storeId=2&storeName=%E5%9B%BD%E5%AE%B6%E5%9F%BA%E7%A1%80%E5%9C%B0%E7%90%86%E4%BF%A1%E6%81%AF%E4%B8%AD%E5%BF%83&fileId=BA420C422A254198BAA5ABAB9CAAFBC1).

The distribution of the three malaria vectors in China is shown in Fig 1. The distribution of *An*. *sinensis* is relatively widespread, with records from the northeast to the southwest, with more dense distribution points in the south. *An*. *lesteri* is mainly found in central and eastern China, with scattered records in the south, presenting a more dispersed spatial pattern. *An*. *minimus* is concentrated in the subtropical and tropical regions south of 33°N in China, including Yunnan, Guizhou, Hainan, Guangxi, Guangdong, Fujian, and Taiwan Provinces.

### Model evaluation and important variables

The results of the model evaluation based on the area under the receiver operating characteristic curve are shown in S1 Fig. The average AUC values of *An*. *lesteri* model, *An*. *minimus* model, and *An*. *sinensis* model were 0.924, 0.962, and 0.906 respectively. All three models fit well. The percent contribution and permutation importance of the variables in each model are shown in Tables 2–4. With these two indicators as the benchmark, variables with any indicator greater than 10% are considered important variables of the model. Monthly precipitation, construction, cropland, elevation, forest, and monthly mean temperature are considered to be important variables in *An*. *lesteri* model. For the *An*. *minimus* model, Monthly precipitation, monthly mean temperature, and elevation are thought to have significant effects on the survival of *An*. *minimus*. The important variables in the *An*. *sinensis* model are Monthly precipitation, construction, monthly mean temperature, and elevation.

**Table 2:** Contribution and Permutation importance of variables in *Anopheles sinensis* model.

Variable	Percent contribution(%)	Permutation importance(%)
pre	56.2	45.3
construction	17.6	8.9
tmp	12.4	26.2
elev	4.7	11.3
cropland	3.2	1.1
forest	2.4	4.4
bare_land	2.4	1.6
gs	0.8	0.9
water	0.4	0.3
wetland	0	0.1
psi	0	0

**Table 3:** Contribution and Permutation importance of variables in *Anopheles lesteri* model.

Variable	Percent contribution(%)	Permutation importance(%)
pre	46.8	57.3
construction	18.9	3.3
cropland	16.9	1.6
elev	8.1	10.1
forest	3.7	10.6
tmp	3.2	10.3
gs	1	1.6
bare_land	1	3.8
water	0.4	1.1
wetland	0.1	0.3
psi	0	0

**Table 4:** Contribution and Permutation importance of variables in *Anopheles minimus* model.

Variable	Percent contribution(%)	Permutation importance(%)
pre	48.3	11.5
tmp	36.8	72
elev	8.4	10.3
construction	3.2	2.7
cropland	1.1	0.9
forest	0.8	0.9
bare_land	0.7	0
gs	0.4	1.5
wetland	0.2	0.2
water	0.1	0.1
psi	0	0

### Habitat suitability map of *Anopheles* mosquitoes and their seasonal variation prediction

**Fig 2:**
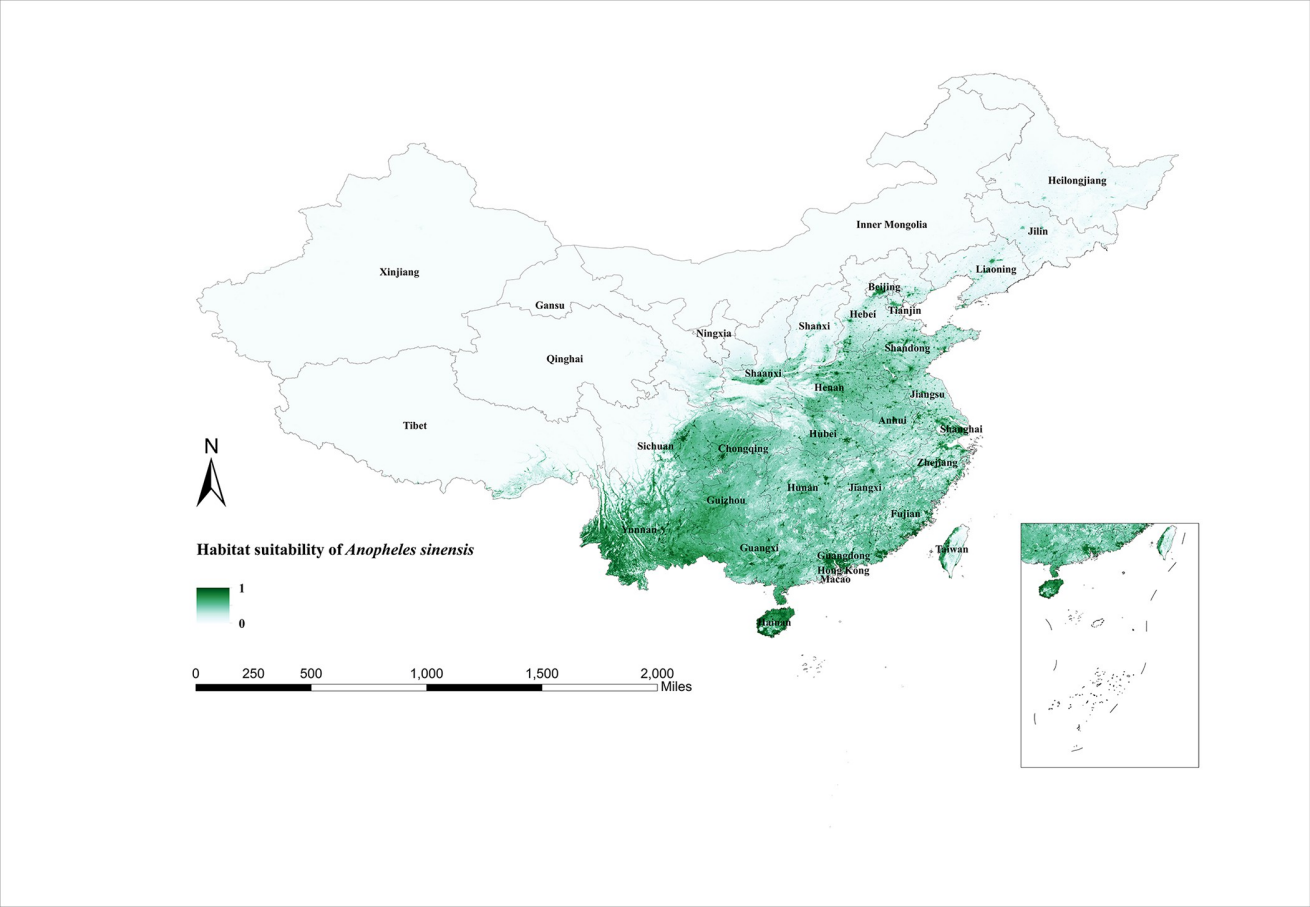
Habitat suitability map for *Anopheles sinensis* in China. The darker colors depict areas of high suitability while lighter colors depict areas of low suitability. The base layer of the map is sourced from the National Catalogue Service For Geographic Information of the Ministry of Natural Resources of the People’s Republic of China (https://www.webmap.cn/mapDataAction.do?method=forw&resType=5&storeId=2&storeName=%E5%9B%BD%E5%AE%B6%E5%9F%BA%E7%A1%80%E5%9C%B0%E7%90%86%E4%BF%A1%E6%81%AF%E4%B8%AD%E5%BF%83&fileId=BA420C422A254198BAA5ABAB9CAAFBC1).

**Fig 3:**
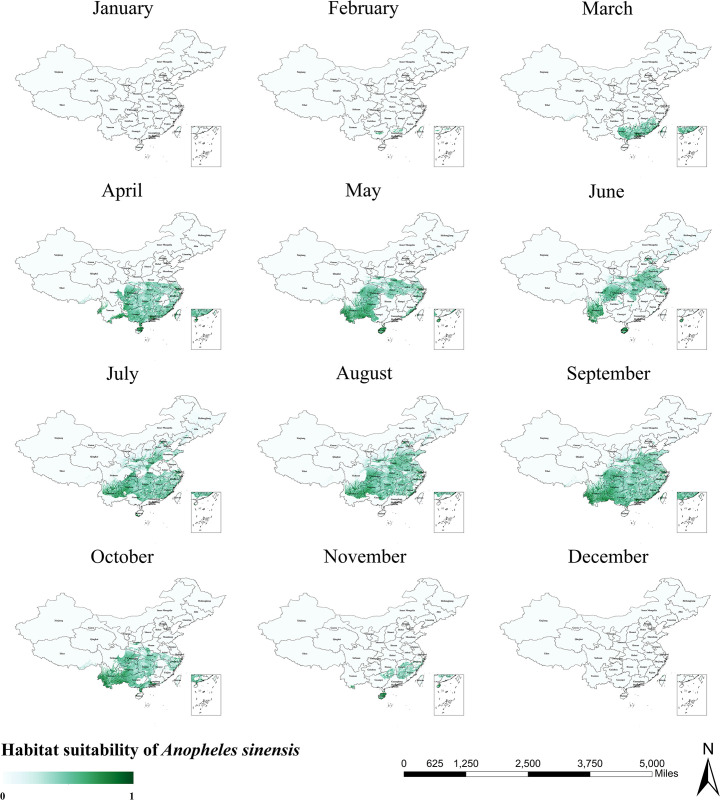
Monthly habitat suitability map for *Anopheles sinensis* in China. The darker colors depict areas of high suitability while lighter colors depict areas of low suitability. The base layer of the map is sourced from the National Catalogue Service For Geographic Information of the Ministry of Natural Resources of the People’s Republic of China (https://www.webmap.cn/mapDataAction.do?method=forw&resType=5&storeId=2&storeName=%E5%9B%BD%E5%AE%B6%E5%9F%BA%E7%A1%80%E5%9C%B0%E7%90%86%E4%BF%A1%E6%81%AF%E4%B8%AD%E5%BF%83&fileId=BA420C422A254198BAA5ABAB9CAAFBC1).

Models reveal that *An*. *sinensis* is active in the southern region of China as well as in the northern region, covering nearly 1/2 of the country (Fig 2). As a whole, high habitat suitability areas are scattered, with concentrations only in Yunnan and Hainan provinces. Fig 3 shows the results of seasonal predictions for *An*. *sinensis*. The distribution range of *An*. *sinensis* in winter is very small, and it was only observed in Taiwan Province (December to February), Guangxi Province (February), and Guangdong Province (February). In March *An*. *sinensis* began to move northwards and by June it covered the areas between the Yangtze and Yellow Rivers, Yunnan Province, and Hainan Province. Over the following three months, the habitat suitability areas for *An*. *sinensis* gradually expanded, peaking in September with widespread distribution over much of the areas south of the Yellow River. The habitat suitability areas for *An*. *sinensis* began to shrink in October and declined even more in November when the predicted graphs were similar to those for March.

**Fig 4:**
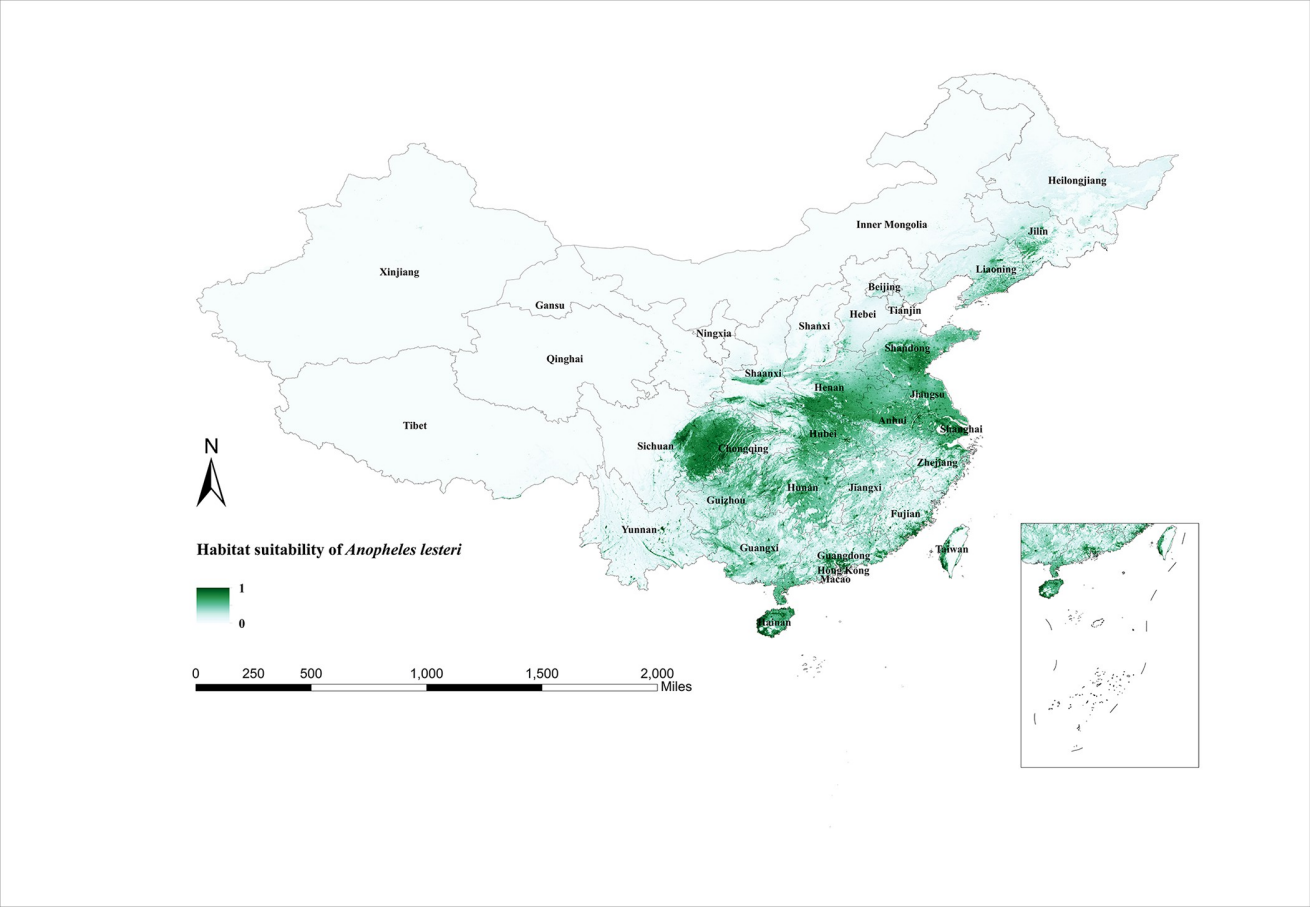
Habitat suitability map for *Anopheles lesteri* in China. The darker colors depict areas of high suitability while lighter colors depict areas of low suitability. The base layer of the map is sourced from the National Catalogue Service For Geographic Information of the Ministry of Natural Resources of the People’s Republic of China (https://www.webmap.cn/mapDataAction.do?method=forw&resType=5&storeId=2&storeName=%E5%9B%BD%E5%AE%B6%E5%9F%BA%E7%A1%80%E5%9C%B0%E7%90%86%E4%BF%A1%E6%81%AF%E4%B8%AD%E5%BF%83&fileId=BA420C422A254198BAA5ABAB9CAAFBC1).

**Fig 5:**
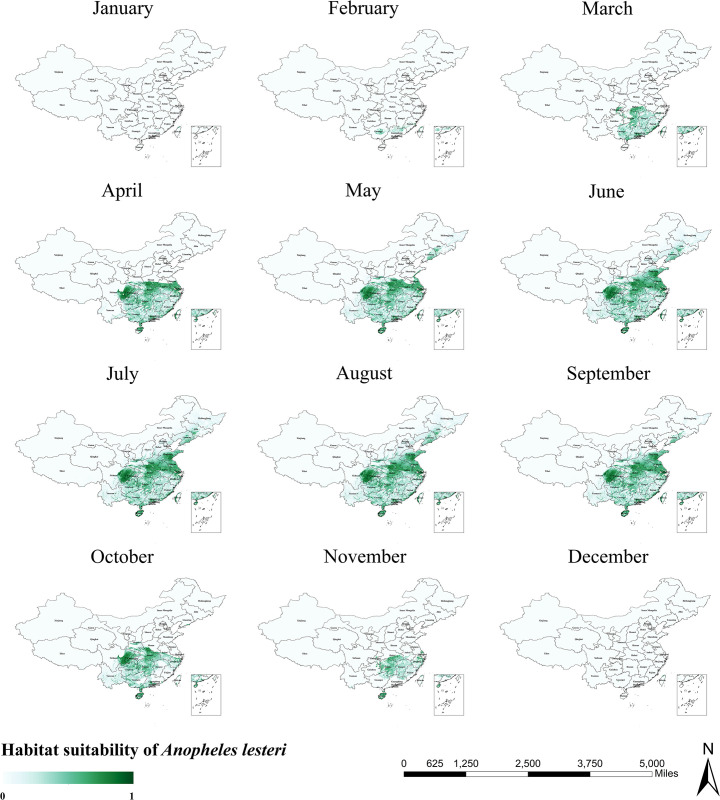
Monthly habitat suitability map for *Anopheles lesteri* in China. The darker colors depict areas of high suitability while lighter colors depict areas of low suitability. The base layer of the map is sourced from the National Catalogue Service For Geographic Information of the Ministry of Natural Resources of the People’s Republic of China (https://www.webmap.cn/mapDataAction.do?method=forw&resType=5&storeId=2&storeName=%E5%9B%BD%E5%AE%B6%E5%9F%BA%E7%A1%80%E5%9C%B0%E7%90%86%E4%BF%A1%E6%81%AF%E4%B8%AD%E5%BF%83&fileId=BA420C422A254198BAA5ABAB9CAAFBC1).

The habitat suitability areas for *An*. *lesteri* are roughly the same as the north-south span of *An*. *sinensis*. The difference is that *An*. *lesteri* is concentrated in the central region, the southeastern coastal region, and Liaoning Province, with more dispersed areas of suitable habitat in the south (Fig 4). Fig 5 clearly illustrates the monthly variation of the habitat suitability maps for *An*. *lesteri*. During the first month of winter (December), *An*. *lesteri* was only visible in Taiwan and Guangxi Provinces. As a result of changes in temperature and precipitation, the suitable areas for *An*. *lesteri* gradually expanded northwards from January to June, reached a yearly maximum in the summer (June to August), and covered more than 21 provinces between 18° and 43°N. After autumn (September to November), *An*. *lesteri* began to migrate southwards and the habitat suitability areas became progressively smaller.

**Fig 6:**
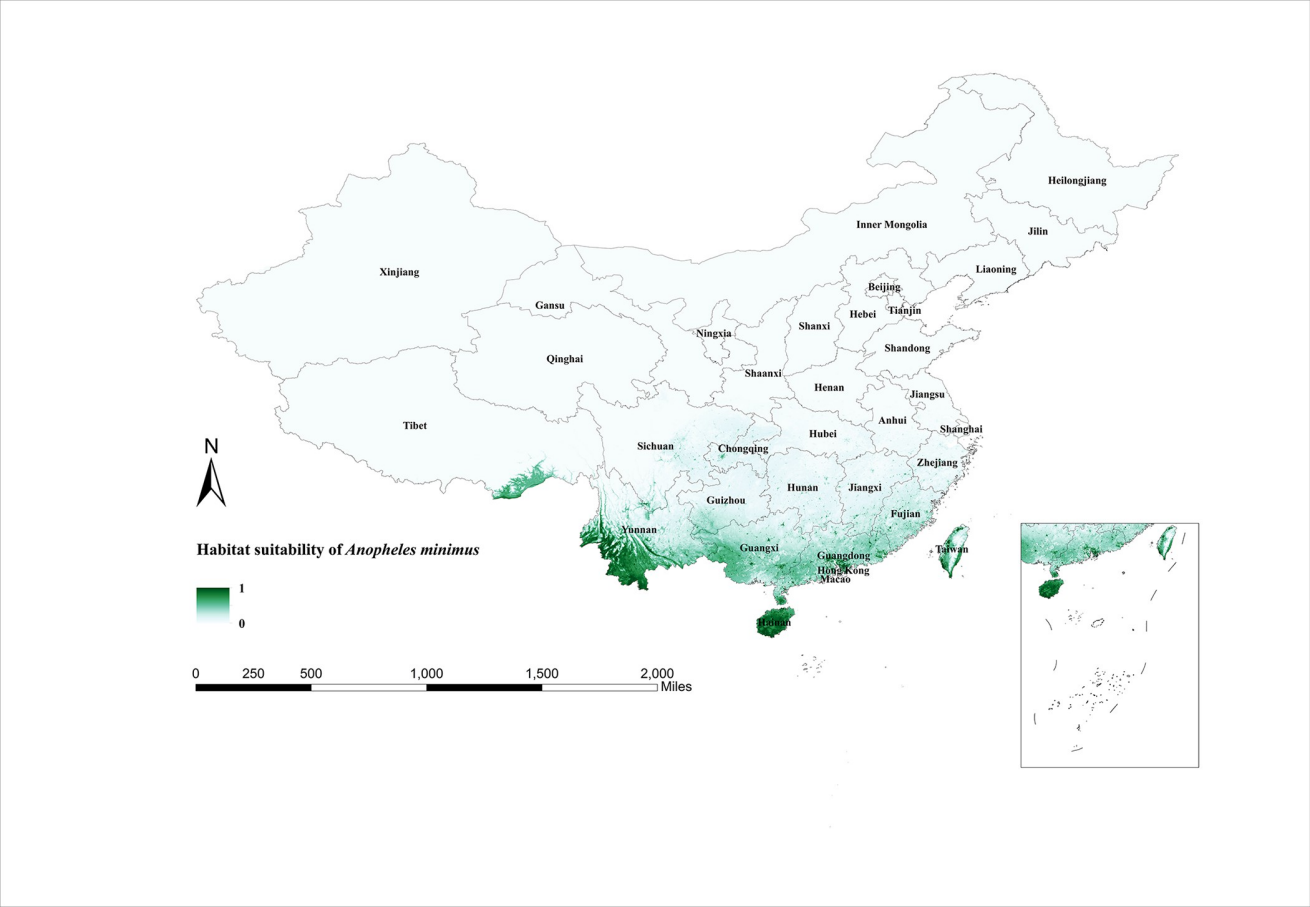
Habitat suitability map for *Anopheles minimus* in China. The darker colors depict areas of high suitability while lighter colors depict areas of low suitability. The base layer of the map is sourced from the National Catalogue Service For Geographic Information of the Ministry of Natural Resources of the People’s Republic of China (https://www.webmap.cn/mapDataAction.do?method=forw&resType=5&storeId=2&storeName=%E5%9B%BD%E5%AE%B6%E5%9F%BA%E7%A1%80%E5%9C%B0%E7%90%86%E4%BF%A1%E6%81%AF%E4%B8%AD%E5%BF%83&fileId=BA420C422A254198BAA5ABAB9CAAFBC1).

**Fig 7:**
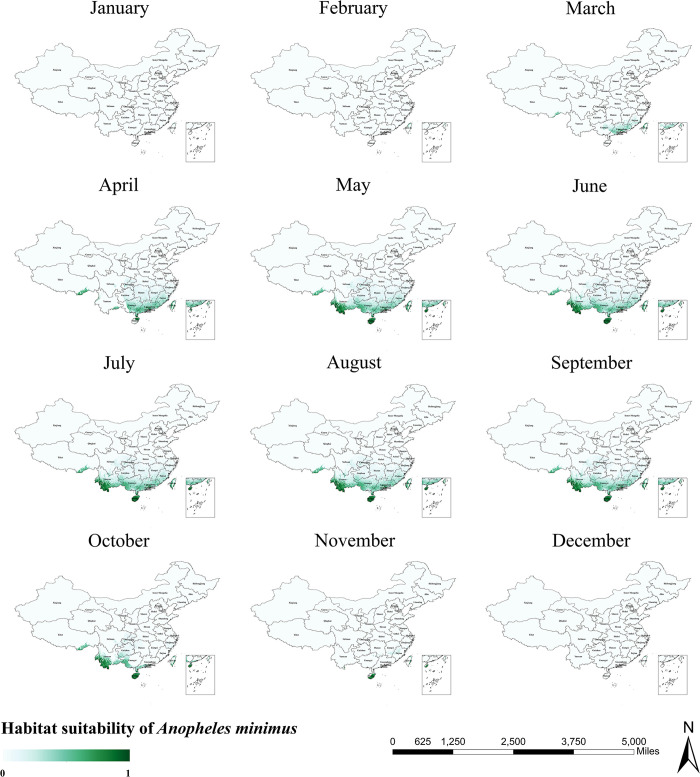
Monthly habitat suitability map for *Anopheles minimus* in China. The darker colors depict areas of high suitability while lighter colors depict areas of low suitability. The base layer of the map is sourced from the National Catalogue Service For Geographic Information of the Ministry of Natural Resources of the People’s Republic of China (https://www.webmap.cn/mapDataAction.do?method=forw&resType=5&storeId=2&storeName=%E5%9B%BD%E5%AE%B6%E5%9F%BA%E7%A1%80%E5%9C%B0%E7%90%86%E4%BF%A1%E6%81%AF%E4%B8%AD%E5%BF%83&fileId=BA420C422A254198BAA5ABAB9CAAFBC1).

The habitat suitability areas predicted by the model for *An*. *minimus* are shown in Fig 6. *An*. *minimus* covers mainly the southern part of China between 18° and 33°N. As with *An*. *sinensis* and *An*. *lesteri*, the habitat suitability areas for *An*. *minimus* in winter are small and confined to the northern part of Taiwan Province (Fig 7). The expansion of the distribution of *An*. *minimus* began in March when it could only be observed in parts of Guangxi Province, Guangdong Province, Jiangxi Province, Fujian Province, and Taiwan Province. After summer, the trend of increasing habitat suitability for *An*. *minimus* flattens out. The southern border provinces of China were very suitable for *An*. *minimus*, including Yunnan, Hainan, Guangxi, Guangdong, Fujian, and Taiwan provinces. After this, the range of *An*. *minimus* began to decrease significantly. By November, only the eastern part of Taiwan Province and the southern part of Hainan Province were suitable for the survival of *An*. *minimus*.

## Discussion

Malaria was once one of the most important insect-borne infectious diseases in China. During its long history of prevalence, it has seriously endangered the health of the Chinese people and constrained social and economic development. After more than 70 years of strict prevention and control, China was officially certified by the World Health Organization to eliminate malaria on June 30, 2021 [43]. The current global malaria epidemic situation remains critical. Although China has successfully achieved the goal of malaria elimination, there are still thousands of imported malaria cases every year [22]. The coexistence of indigenous malaria vectors and imported malaria from abroad will continue for a considerable period. The results of vector monitoring showed that from 2011 to 2020 [22], the population density of *An*. *sinensis* accounted for 94.78% of the population, the most widely distributed. *An*. *lesteri* was identified in Guizhou, Sichuan, Hainan, and Liaoning Provinces, *An*. *minimus* was identified in Yunnan and Hainan Provinces, and *An*. *dirus* was identified only in Hainan Province. Vector monitoring may be limited by terrain, weather, and other constraints, and the results do not adequately reflect the distribution of malaria vectors. The use of ecological niche modeling to explore the current distribution of malaria vectors and seasonal changes in China can provide a powerful complement to vector monitoring and an important reference for the development of integrated vector control programs.

Through a detailed review of the literature in recent years, we obtained new sampling records for the three vectors. The aggregated results are shown in Fig 1. The distribution range of *An*. *minimus* was little changed from that reported in previous studies [25,31]. The prevalence area of *An*. *lesteri* has decreased, being particularly severe in Yunnan, Fujian, Zhejiang, and Anhui provinces, probably due to malaria control and environmental changes in recent years [44,45]. The northernmost location of *An*. *sinensis* has been extended to Heilongjiang Province (around 49°N), further expanding its range. This is consistent with simulations by Ren et al. that climate change will lead to the increase of climate-suitable areas of *An*. *sinensis* in northern China [31].

Using new sampling records, as well as climatic and topographic data, ecological niche models were developed for the three malaria vectors. Tables 2–4 and S2–S4 Figs show the important variables of the model and the rough trends in their influence on the occurrence probability of *Anopheles* mosquitoes. In terms of contribution and permutation importance, both monthly precipitation and mean monthly temperature play crucial roles in the distribution of the three species of *Anopheles*. Temperature affects the physiology of mosquitoes, including development speed, breeding behavior, and mortality [46]. Precipitation provides mosquitoes with the water necessary for their larval and pupae habitats, and their presence is strongly regulated by (usually seasonal) rainfall [47,48]. Warm and suitable temperatures and high humidity effectively extend the life span of adult mosquitoes and shorten their blood-sucking intervals, leading to faster transmission of the virus [49].

In addition to climatic variables, topographic variables also influence the distribution of *Anopheles* mosquitoes to some extent. The variable response curves show that all three species of *Anopheles* favor lower altitudes, indicating that they are better suited to survive in areas of lower terrain (S2–S4 Figs). The higher the altitude, the lower the density and humidity of the air, and the lower the temperature, conditions that are not conducive to the development and breeding of *Anopheles* mosquitoes [50]. For land cover variables, construction promoted the occurrence of *An*. *sinensis* and *An*. *lesteri* (S2 and S3 Figs). Construction is in densely populated and active areas such as cities or villages, where the presence of humans and livestock attracts *Anopheles* mosquitoes to feed. The probability of occurrence of *An*. *lesteri* was positively correlated with the cropland (S3 Fig) because the crops and small reservoirs within the cropland provide suitable habitats for *An*. *lesteri* to breed. However, the forest has a negative impact on the occurrence of *An*. *lesteri* (S3 Fig), which is contrary to the previous research [51]. Wang et al. pointed out that while rice fields and forests constitute the natural habitat of mosquitoes, the role of human settlements for individual species is significant [52]. It is speculated that the reason why the forest is not conducive to the survival of *An*. *lesteri* may be that the plants are too luxuriant, the people are rarely visited, and the *An*. *lesteri* lacks blood food for survival.

Standard deviation maps of habitat suitability for the three species of *Anopheles* show little difference between the replicated models, indicating that the models are relatively robust (S5–S7 Figs). The predictions from the model output reveal that the most widespread malaria vector in China remains *An*. *sinensis*, with a wide region from the northeast (Heilongjiang province) to the southwest (Yunnan province) suitable for its survival. The second is the *An*. *lesteri*, with suitable habitat areas concentrated in the central, eastern, and southern regions of China. The suitable habitat areas of *An*. *minimus* are the smallest, which are only distributed in the border provinces of southern China. From 2011 to 2020, malaria cases were reported in all provinces of China, mainly in the provinces of Yunnan, Guangxi, Jiangsu, Sichuan and Henan [22]. These regions are all located in areas of high habitat suitability for malaria vectors and will have a high risk of local transmission in the event of a malaria case.

As precipitation and temperature are important climatic factors influencing mosquito population dynamics, we explored seasonal trends in the distribution range of *Anopheles* mosquitoes based on the habitat suitability maps output by the maxent model, using fluctuations in the mean monthly temperature and monthly precipitation to obtain monthly habitat suitability maps for three species of *Anopheles* (Figs 3, 5 and 7). Taken together, the monthly trends in the distribution ranges of the three species of *Anopheles* from January to December were similar, presenting a general pattern of moving from south to north with a gradual increase in area, and then moving from north to south with a gradual decrease in area. However, the habitat suitability areas of the three species of *Anopheles* peaked at different times, with *An*. *lesteri* and *An*. *minimus* in the summer (June-August) and *An*. *sinensis* in the autumn (September). This phenomenon is caused by the difference in the suitable temperature and precipitation of the three species of *Anopheles*. One area worthy of attention is Liaoning Province in Northeast China. During the malaria pandemic, the peak months of the season in Liaoning Province mostly fluctuate between July and August [53]. From our prediction map, it is concluded that *Anopheles* mosquitoes (*An*. *lesteri* and *An*. *sinensis*) reaches its seasonal peak in Liaoning Province from June to August, which is generally consistent with the epidemiological pattern of malaria in the province.

There are certain limitations to our study. First of all, this study did not consider the interaction between organisms. In nature, the *Anopheles* mosquito has a predatory or competitive relationship with other species in their range (e.g. fish and shrimps). It is a biological instinct to avoid harm, so mosquitoes rarely survive in habitats with predators and competitors [54,55]. Second, official monitoring data are confidential, so all *Anopheles* mosquito occurrence data used in our study were obtained from journal publications. The *Anopheles* mosquito occurrence data used in our modeling may not be comprehensive, leading to a possible underestimation of habitat suitability areas for *Anopheles* mosquitoes. Another point is that human distribution data were not included in the modeling process. As a source of blood meal for mosquitoes, the distribution of human populations may be influencing the growth of vector populations and thus the spread of disease [56]. A lack of this data may underestimate the probability of the presence of *Anopheles* mosquitoes in areas with high human density and make it difficult to assess the specific impact of human activities on the distribution of *Anopheles* mosquitoes. In addition to human blood, the three species of *Anopheles* also feed on the blood of livestock, foraging mainly in human houses and livestock barns [57]. Although we used the construction variable, which can roughly reflect the distribution of human houses and livestock barns. However, this may still lead to some bias in the prediction results. In future work, joint species distribution models could be considered to explore how the interactions between *Anopheles* mosquito and other species affect their distribution and to include a population density map to assess the human contribution to *Anopheles* mosquito population growth or decline. In addition, linking historical disease data to vector data may be more meaningful than simply predicting the distribution of vectors. Where data allow, the addition of detailed information on imported malaria cases helps to further accurately model the risk of the spread of imported malaria in China.

## Conclusion

In addition to precipitation and temperature, which are important variables affecting the distribution of *Anopheles* mosquitoes, topographic variables also have an impact on the distribution of *Anopheles* mosquitoes. *An*. *sinensis* is the most widespread malaria vector in China, with a wide region from the northeast (Heilongjiang Province) to the southwest (Yunnan Province) suitable for its survival. Suitable habitat areas for *An*. *lesteri* are concentrated in the central, eastern, and southern regions of China. The suitable habitat areas of *An*. *minimus* are the smallest and are only distributed in the border provinces of southern China. Based on the results of the ecological niche model, we further assessed the current seasonal variation in habitat suitability areas for the three major malaria vectors in China. Considering that resources for vector monitoring may be limited, the results of this study may provide some reference for vector control strategies to optimize the use of resources to address the risk of re-transmission of imported malaria.

## Supporting information

S1 Fig Results of the model evaluation based on the area under the receiver operating characteristic curve. The curves show the mean ROC of the 10 replicate maxent runs (red) and the mean +/- one standard deviation (blue). The black line indicates random prediction. (a) *Anopheles lesteri* model. (b) *Anopheles minimus* model. (c) *Anopheles sinensis* model.
(TIF)

S2 Fig Response curves for important variables in the *Anopheles sinensis* model. The curves show the mean response of the 10 replicate maxent runs (red) and the mean +/- one standard deviation (blue). (a) pre. (b) construction. (c) tmp. (d) elev.
(TIF)

S3 Fig Response curves for important variables in the *Anopheles lesteri* model. The curves show the mean response of the 10 replicate maxent runs (red) and the mean +/- one standard deviation (blue). (a) pre. (b) construction. (c) cropland. (d) elev. (e) forest. (f) tmp.
(TIF)

S4 Fig Response curves for important variables in the *Anopheles minimus* model. The curves show the mean response of the 10 replicate maxent runs (red) and the mean +/- one standard deviation (blue). (a) pre. (b) tmp. (c) elev.
(TIF)

S5 Fig Standard deviation map of habitat suitability for *Anopheles sinensis*. The map shows the standard deviation of output rasters of 10 repeated maxent runs. The darker colors depict areas of high standard deviation while lighter colors depict areas of low standard deviation. The base layer of the map is sourced from the National Catalogue Service For Geographic Information of the Ministry of Natural Resources of the People’s Republic of China (https://www.webmap.cn/mapDataAction.do?method=forw&resType=5&storeId=2&storeName=%E5%9B%BD%E5%AE%B6%E5%9F%BA%E7%A1%80%E5%9C%B0%E7%90%86%E4%BF%A1%E6%81%AF%E4%B8%AD%E5%BF%83&fileId=BA420C422A254198BAA5ABAB9CAAFBC1).
(TIF)

S6 Fig Standard deviation map of habitat suitability for *Anopheles lesteri*. The map shows the standard deviation of output rasters of 10 repeated maxent runs. The darker colors depict areas of high standard deviation while lighter colors depict areas of low standard deviation. The base layer of the map is sourced from the National Catalogue Service For Geographic Information of the Ministry of Natural Resources of the People’s Republic of China (https://www.webmap.cn/mapDataAction.do?method=forw&resType=5&storeId=2&storeName=%E5%9B%BD%E5%AE%B6%E5%9F%BA%E7%A1%80%E5%9C%B0%E7%90%86%E4%BF%A1%E6%81%AF%E4%B8%AD%E5%BF%83&fileId=BA420C422A254198BAA5ABAB9CAAFBC1).
(TIF)

S7 Fig Standard deviation map of habitat suitability for *Anopheles minimus*. The map shows the standard deviation of output rasters of 10 repeated maxent runs. The darker colors depict areas of high standard deviation while lighter colors depict areas of low standard deviation. The base layer of the map is sourced from the National Catalogue Service For Geographic Information of the Ministry of Natural Resources of the People’s Republic of China (https://www.webmap.cn/mapDataAction.do?method=forw&resType=5&storeId=2&storeName=%E5%9B%BD%E5%AE%B6%E5%9F%BA%E7%A1%80%E5%9C%B0%E7%90%86%E4%BF%A1%E6%81%AF%E4%B8%AD%E5%BF%83&fileId=BA420C422A254198BAA5ABAB9CAAFBC1).
(TIF)

S1 Table Coordinates of distribution points of *Anopheles* mosquitoes after purification based on ENMTools software. (XLSX)

## References

[1] Huang F, Zhang L, Xue J-B, Zhou H-N, Thi A, Zhang J, et al. From control to elimination: a spatial-temporal analysis of malaria along the China-Myanmar border. Infectious Diseases of Poverty. 2020;9:1–13. 33213516 10.1186/s40249-020-00777-1 PMC7676414

[2] Cowman AF, Healer J, Marapana D, Marsh K. Malaria: biology and disease. Cell. 2016;167(3):610–24. doi: 10.1016/j.cell.2016.07.055 27768886

[3] Miller LH, Baruch DI, Marsh K, Doumbo OK. The pathogenic basis of malaria. Nature. 2002;415(6872):673–9. doi: 10.1038/415673a 11832955

[4] WHO. World malaria report 2022: World Health Organization; 2022.

[5] Huang F, Feng X-Y, Zhou S-S, Tang L-H, Xia Z-G. Establishing and applying an adaptive strategy and approach to eliminating malaria: practice and lessons learnt from China from 2011 to 2020. Emerging Microbes & Infections. 2022;11(1):314–25. doi: 10.1080/22221751.2022.2026740 34989665 PMC8786258

[6] Zu-Jie Z. The malaria situation in the People’s Republic of China. Bulletin of the World Health Organization. 1981;59(6):931. 6978199 PMC2396122

[7] Tang L. Chinese achievements in malaria control and research. Chin J Parasitol Parasit Dis. 1999;17(5):27–9. 12563848

[8] Zhou Z, editor Current status of malaria in China. The Asia and Pacific Conference on Malaria; 1985.

[9] China MoHotPsRo. Action plan of China malaria elimination (2010–2020). 2010.

[10] Feng J, Tu H, Zhang L, Zhang S, Jiang S, Xia Z, et al. Mapping transmission foci to eliminate malaria in the People’s Republic of China, 2010–2015: a retrospective analysis. BMC infectious diseases. 2018;18:1–10. 29514598 10.1186/s12879-018-3018-8 PMC5840925

[11] Feng J, Zhang L, Huang F, Yin J-H, Tu H, Xia Z-G, et al. Ready for malaria elimination: zero indigenous case reported in the People’s Republic of China. Malaria journal. 2018;17:1–13. 30157876 10.1186/s12936-018-2444-9 PMC6116478

[12] Hu T, Liu Y-B, Zhang S-S, Xia Z-G, Zhou S-S, Yan J, et al. Shrinking the malaria map in China: measuring the progress of the National Malaria Elimination Programme. Infectious Diseases of Poverty. 2016;5:1–7. 27197517 10.1186/s40249-016-0146-5 PMC4873993

[13] WHO. World Malaria Report 2018: World Health Organization; 2018.

[14] Cao J, Newby G, Cotter C, Hsiang MS, Larson E, Tatarsky A, et al. Achieving malaria elimination in China. The Lancet Public Health. 2021;6(12):e871–e2. doi: 10.1016/S2468-2667(21)00201-2 34838192 PMC9022785

[15] Zhang L, Yi B, Xia Z, Yin J. Epidemiological characteristics of malaria in China, 2021. Chinese Journal of Parasitology and Parasitic Diseases. 2022;40(02):135–9.

[16] Zhang L, Yi B, Yin J, Xia Z. Epidemiological characteristics of malaria in China,2022. Chinese Journal of Parasitology and Parasitic Diseases. 2023;41(02):137–41.

[17] Zhang L, Feng J, Tu H, Yin J, Xia Z. Malaria epidemiology in China in 2020. Chinese Journal of Parasitology and Parasitic Diseases. 2021;39(02):195–9.

[18] Zhang L, Feng J, Xia Z, Zhou S. Epidemiological characteristics of malaria and progress on its elimination in China in 2019. Chinese Journal of Parasitology and Parasitic Diseases. 2020;38(02):133–8.

[19] Zhang L, Feng J, Zhang S, Xia Z, Zhou S. Epidemiological characteristics of malaria and the progress towards its elimination in China in 2018. Chinese Journal of Parasitology and Parasitic Diseases. 2019;37(02):241–7.

[20] Zhang L, Feng J, Zhang S, Xia Z, Zhou S. The progress of national malaria elimination and epidemiological characteristics of malaria in China in 2017. Chinese Journal of Parasitology and Parasitic Diseases. 2018;36(03):201–9.

[21] Danis K, Baka A, Lenglet A, Van Bortel W, Terzaki I, Tseroni M, et al. Autochthonous Plasmodium vivax malaria in Greece, 2011. Eurosurveillance. 2011;16(42):19993. 22027375

[22] Xia Z, Feng J, Zhang L, Feng X, Huang F, Yin J, et al. Achieving malaria elimination in China: analysis on implementation and effectiveness of the surveillance-response system. Chinese Journal of Parasitology and Parasitic Diseases. 2021;39(06):733–41.

[23] CDC C. National Malaria Elimination Monitoring Program (2015 version): Chinese Center for Disease Control and Prevention 2015.

[24] Feng X, Zhang L, Feng J, Zhi-gui X, Ning X. Analysis of national malaria surveillance in China in 2013. J Path Biol. 2014;9:1117–20.

[25] Zhang S, Guo S, Feng X, Afelt A, Frutos R, Zhou S, et al. Anopheles vectors in mainland China while approaching malaria elimination. Trends in Parasitology. 2017;33(11):889–900. doi: 10.1016/j.pt.2017.06.010 28734898

[26] Ma A, Wang J, Wang D, Ren Z. Prediction of potential distribution of Anopheles sinensis in China based on MaxEnt. Chin J Vector Biol Control. 2014;25:393–8.

[27] Ren Z, Wang D, Hwang J, Bennett A, Sturrock HJ, Ma A, et al. Spatial-temporal variation and primary ecological drivers of Anopheles sinensis human biting rates in malaria epidemic-prone regions of China. PLoS One. 2015;10(1):e0116932. doi: 10.1371/journal.pone.0116932 25611483 PMC4303435

[28] Prevention MoHD, Bureau C. Handbook for malaria control and prevention. People’s Hygiene Publishing House Press Beijing; 2007.

[29] Feng X, Feng J, Zhang L, Tu H, Xia Z. Vector control in China, from malaria endemic to elimination and challenges ahead. Infectious Diseases of Poverty. 2022;11(1):1–11. 35562786 10.1186/s40249-022-00971-3 PMC9102289

[30] Xu L-S, Wu J-J, Xu B-H, Li L-S. Surveillance on malaria in residual region of Anopheles anthropophagus in Fujian province, China. Chinese Journal of Parasitology and Parasitic Diseases. 2004;17:28–30.

[31] Ren Z, Wang D, Ma A, Hwang J, Bennett A, Sturrock HJ, et al. Predicting malaria vector distribution under climate change scenarios in China: challenges for malaria elimination. Scientific reports. 2016;6(1):1–13. 26868185 10.1038/srep20604 PMC4751525

[32] Warren DL, Glor RE, Turelli M. ENMTools: a toolbox for comparative studies of environmental niche models. Ecography. 2010;33(3):607–11.

[33] Outammassine A, Zouhair S, Loqman S. Global potential distribution of three underappreciated arboviruses vectors (Aedes japonicus, Aedes vexans and Aedes vittatus) under current and future climate conditions. Transboundary and Emerging Diseases. 2022;69(4):e1160–e71. doi: 10.1111/tbed.14404 34821477

[34] Hertig E. Distribution of Anopheles vectors and potential malaria transmission stability in Europe and the Mediterranean area under future climate change. Parasites & vectors. 2019;12:1–9. doi: 10.1186/s13071-018-3278-6 30621785 PMC6325871

[35] Liu B, Gao X, Ma J, Jiao Z, Xiao J, Hayat MA, et al. Modeling the present and future distribution of arbovirus vectors Aedes aegypti and Aedes albopictus under climate change scenarios in Mainland China. Science of the Total Environment. 2019;664:203–14. doi: 10.1016/j.scitotenv.2019.01.301 30743113

[36] Peng S. 1-km Monthly Mean Temperature Dataset for China (1901–2021). National Tibetan Plateau Data Center. 2019.

[37] Peng S. 1-km monthly precipitation dataset for China (1901–2021). National Tibetan Plateau Data Center. 2020.

[38] Liu Y, Wang H, Wang Y, Cai L. China‘s land cover fraction dataset (2001–2018). National Tibetan Plateau Data Center. 2021.

[39] Kaky E, Nolan V, Alatawi A, Gilbert F. A comparison between Ensemble and MaxEnt species distribution modelling approaches for conservation: A case study with Egyptian medicinal plants. Ecological Informatics. 2020;60:101150.

[40] Thibaud E, Petitpierre B, Broennimann O, Davison AC, Guisan A. Measuring the relative effect of factors affecting species distribution model predictions. Methods in Ecology and Evolution. 2014;5(9):947–55.

[41] Elith J, Phillips SJ, Hastie T, Dudík M, Chee YE, Yates CJ. A statistical explanation of MaxEnt for ecologists. Diversity and distributions. 2011;17(1):43–57.

[42] Peterson AT. Uses and requirements of ecological niche models and related distributional models. Biodiversity informatics. 2006;3.

[43] WHO. From 30 million cases to zero: China is certified malaria-free by WHO. World Health Organization. 2021.

[44] Xu B, Xu Ls, Zhang S, Yang F. Evaluation and surveillance on the effect of control for the Anopheles anthropophagus in Fujian province. Chinese Journal of Zoonoses. 2009;23:5.

[45] Chen G, Li W, Ren Z, Yang S. Distribution of Anopheles anthropophagus in Yunnan Province and the characteristics of changes after control. Chinese Journal of Parasitic Diseases Control. 2004;2:53.

[46] Patz JA, Martens W, Focks DA, Jetten TH. Dengue fever epidemic potential as projected by general circulation models of global climate change. Environmental health perspectives. 1998;106(3):147–53. doi: 10.1289/ehp.98106147 9452414 PMC1533051

[47] Chemison A, Ramstein G, Tompkins AM, Defrance D, Camus G, Charra M, et al. Impact of an accelerated melting of Greenland on malaria distribution over Africa. Nature Communications. 2021;12(1):3971. doi: 10.1038/s41467-021-24134-4 34172729 PMC8233338

[48] Mutheneni SR, Morse AP, Caminade C, Upadhyayula SM. Dengue burden in India: recent trends and importance of climatic parameters. Emerging microbes & infections. 2017;6(1):1–10. doi: 10.1038/emi.2017.57 28790459 PMC5583666

[49] Tseng W-C, Chen C-C, Chang C-C, Chu Y-H. Estimating the economic impacts of climate change on infectious diseases: a case study on dengue fever in Taiwan. Climatic Change. 2009;92(1–2):123–40.

[50] West JB. Commuting to high altitude: value of oxygen enrichment of room air. High Altitude Medicine & Biology. 2002;3(2):223–35. 10.1089/15270290260131948 12162865

[51] Liu H, Zhou Y, Deng Y, Lin Z, Zhang C, Chen Q, et al. Malaria from hyperendemicity to elimination along international borders in Yunnan, China during 2003–2020: a case study. Infectious Diseases of Poverty. 2022;11(03):69–80. 35538510 10.1186/s40249-022-00972-2 PMC9088148

[52] Wang T, Fan Z-W, Ji Y, Chen J-J, Zhao G-P, Zhang W-H, et al. Mapping the distributions of mosquitoes and mosquito-borne arboviruses in China. Viruses. 2022;14(4):691. doi: 10.3390/v14040691 35458421 PMC9031751

[53] Anonymous. A preliminary study on the epidemiological pattern of malaria in Liaoning Province. Liaoning Intermediate Medical Journal. 1978;5:26–7.

[54] Muturi EJ, Mwangangi J, Shililu J, Jacob BG, Mbogo C, Githure J, et al. Environmental factors associated with the distribution of Anopheles arabiensis and Culex quinquefasciatus in a rice agro-ecosystem in Mwea, Kenya. Journal of Vector Ecology. 2008;33(1):56–63. 18697307 10.3376/1081-1710(2008)33[56:efawtd]2.0.co;2

[55] Gouagna LC, Rakotondranary M, Boyer S, Lempérière G, Dehecq J-S, Fontenille D. Abiotic and biotic factors associated with the presence of Anopheles arabiensis immatures and their abundance in naturally occurring and man-made aquatic habitats. Parasites & vectors. 2012;5(1):1–12. doi: 10.1186/1756-3305-5-96 22608179 PMC3461495

[56] Ho S, Speldewinde P, Cook A. Predicting arboviral disease emergence using Bayesian networks: a case study of dengue virus in Western Australia. Epidemiology & Infection. 2017;145(1):54–66. doi: 10.1017/S0950268816002090 27620510 PMC9507333

[57] Fu W, Yan Z, Guo J, Chen B. Research progress of the genus Anopheles of mosquitoes in China. Chinese Journal of Vector Biology And Control. 2021;32(5):519–25.

