# Supplementary figures and images for: Seasonal prediction of the distribution of three major malaria vectors in China: Based on an ecological niche model
Source: PLoS Negl Trop Dis. 2024 Jan 18;18(1):e0011884. doi: 10.1371/journal.pntd.0011884 (PMC10796015; doi:10.1371/journal.pntd.0011884)

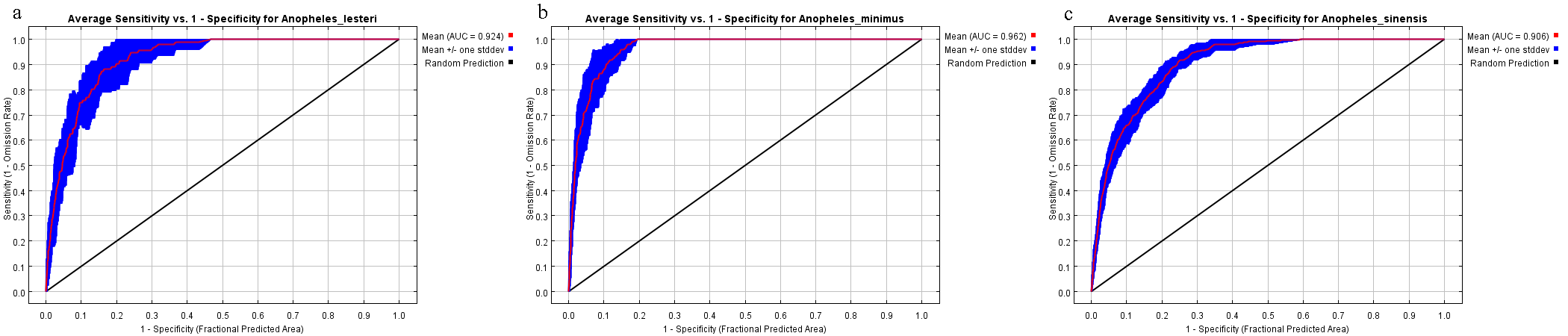

Supplement: S1 Fig — The curves show the mean ROC of the 10 replicate maxent runs (red) and the mean +/- one standard deviation (blue). The black line indicates random prediction. (a) Anopheles lesteri model. (b) Anopheles minimus model. (c) Anopheles sinensis model. (TIF) [file pntd.0011884.s001.tif]

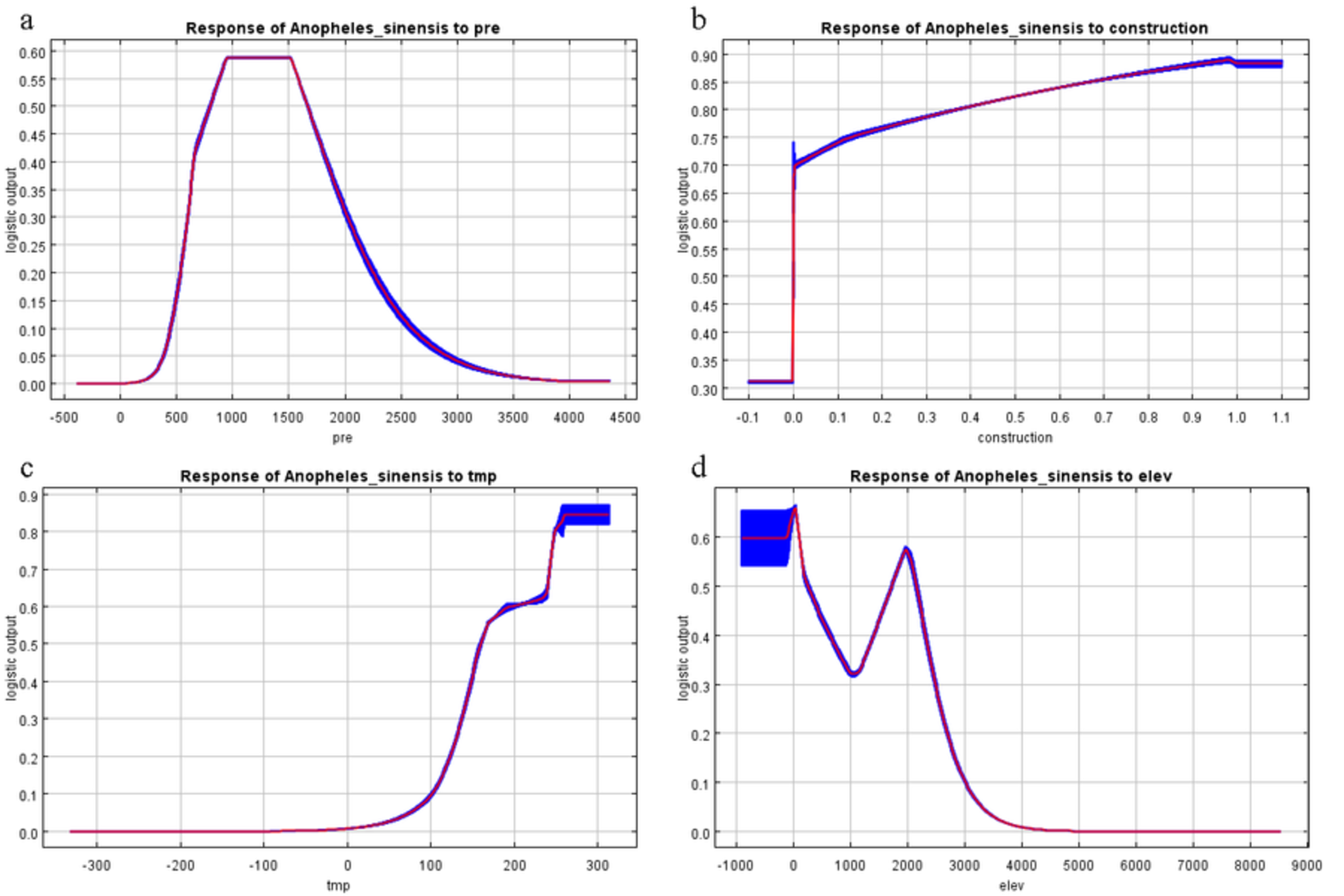

Supplement: S2 Fig — The curves show the mean response of the 10 replicate maxent runs (red) and the mean +/- one standard deviation (blue). (a) pre. (b) construction. (c) tmp. (d) elev. (TIF) [file pntd.0011884.s002.tif]

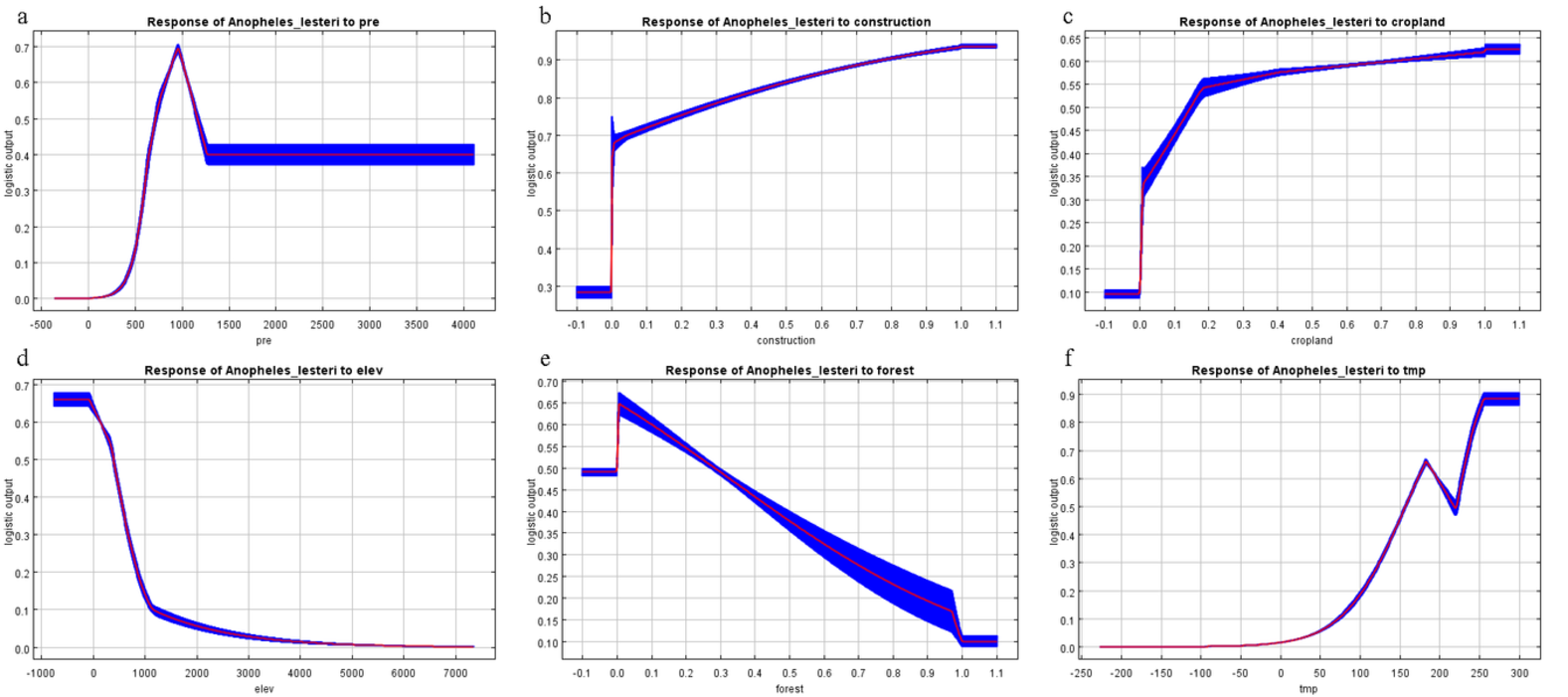

Supplement: S3 Fig — The curves show the mean response of the 10 replicate maxent runs (red) and the mean +/- one standard deviation (blue). (a) pre. (b) construction. (c) cropland. (d) elev. (e) forest. (f) tmp. (TIF) [file pntd.0011884.s003.tif]

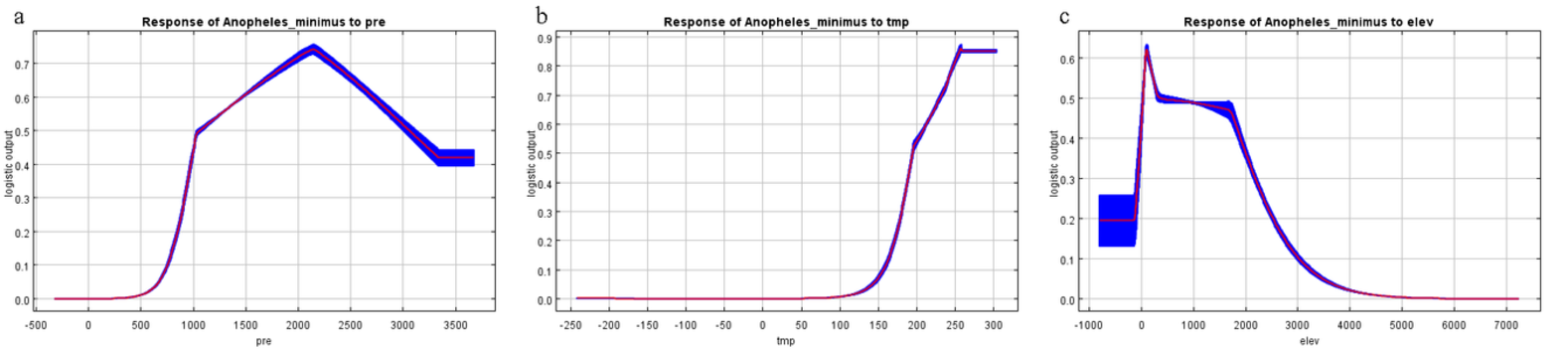

Supplement: S4 Fig — The curves show the mean response of the 10 replicate maxent runs (red) and the mean +/- one standard deviation (blue). (a) pre. (b) tmp. (c) elev. (TIF) [file pntd.0011884.s004.tif]

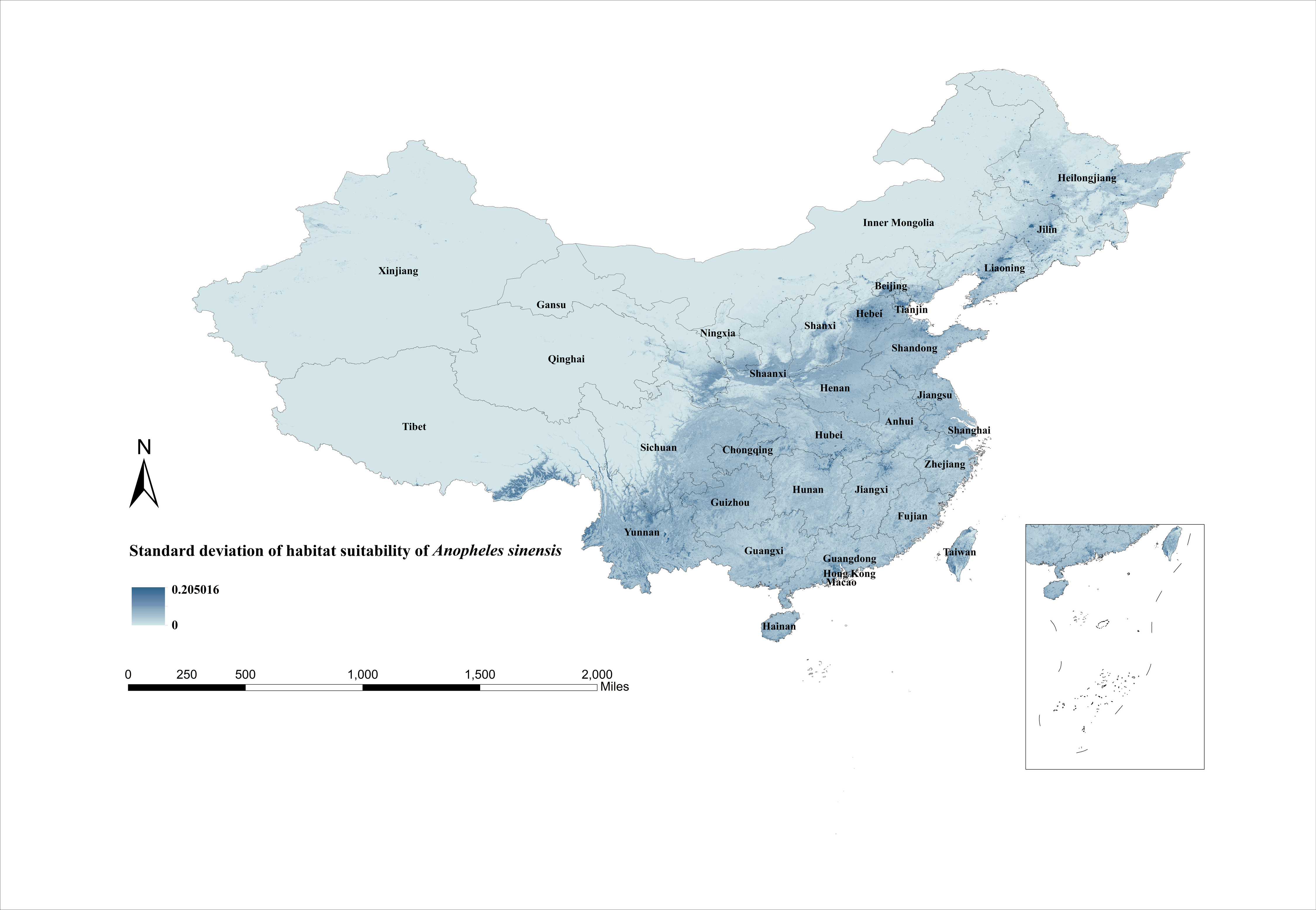

Supplement: S5 Fig — The map shows the standard deviation of output rasters of 10 repeated maxent runs. The darker colors depict areas of high standard deviation while lighter colors depict areas of low standard deviation. The base layer of the map is sourced from the National Catalogue Service For Geographic Information of the Ministry of Natural Resources of the People’s Republic of China (https://www.webmap.cn/mapDataAction.do?method=forw&resType=5&storeId=2&storeName=%E5%9B%BD%E5%AE%B6%E5%9F%BA%E7%A1%80%E5%9C%B0%E7%90%86%E4%BF%A1%E6%81%AF%E4%B8%AD%E5%BF%83&fileId=BA420C422A254198BAA5ABAB9CAAFBC1). (TIF) [file pntd.0011884.s005.tif]

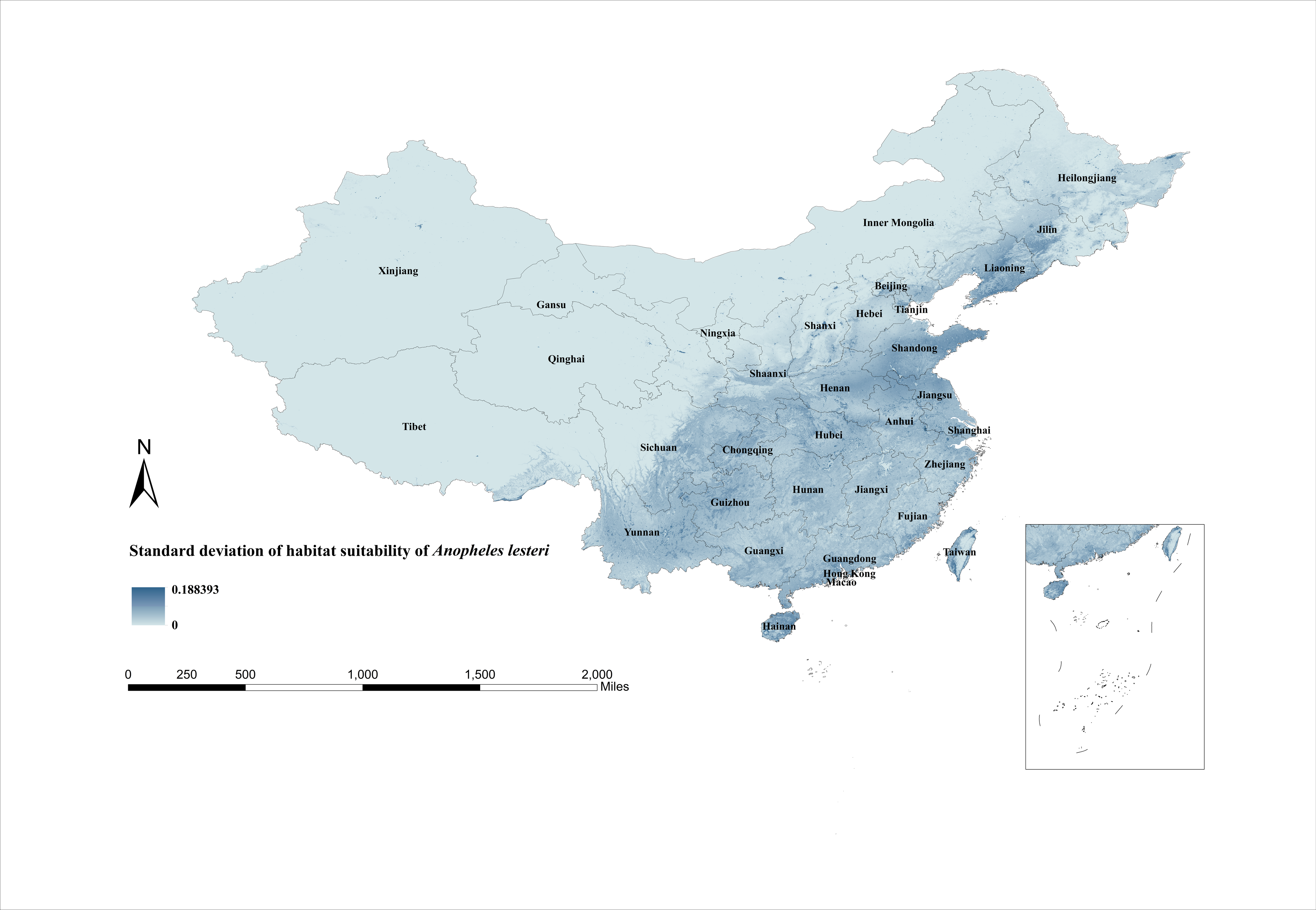

Supplement: S6 Fig — The map shows the standard deviation of output rasters of 10 repeated maxent runs. The darker colors depict areas of high standard deviation while lighter colors depict areas of low standard deviation. The base layer of the map is sourced from the National Catalogue Service For Geographic Information of the Ministry of Natural Resources of the People’s Republic of China (https://www.webmap.cn/mapDataAction.do?method=forw&resType=5&storeId=2&storeName=%E5%9B%BD%E5%AE%B6%E5%9F%BA%E7%A1%80%E5%9C%B0%E7%90%86%E4%BF%A1%E6%81%AF%E4%B8%AD%E5%BF%83&fileId=BA420C422A254198BAA5ABAB9CAAFBC1). (TIF) [file pntd.0011884.s006.tif]

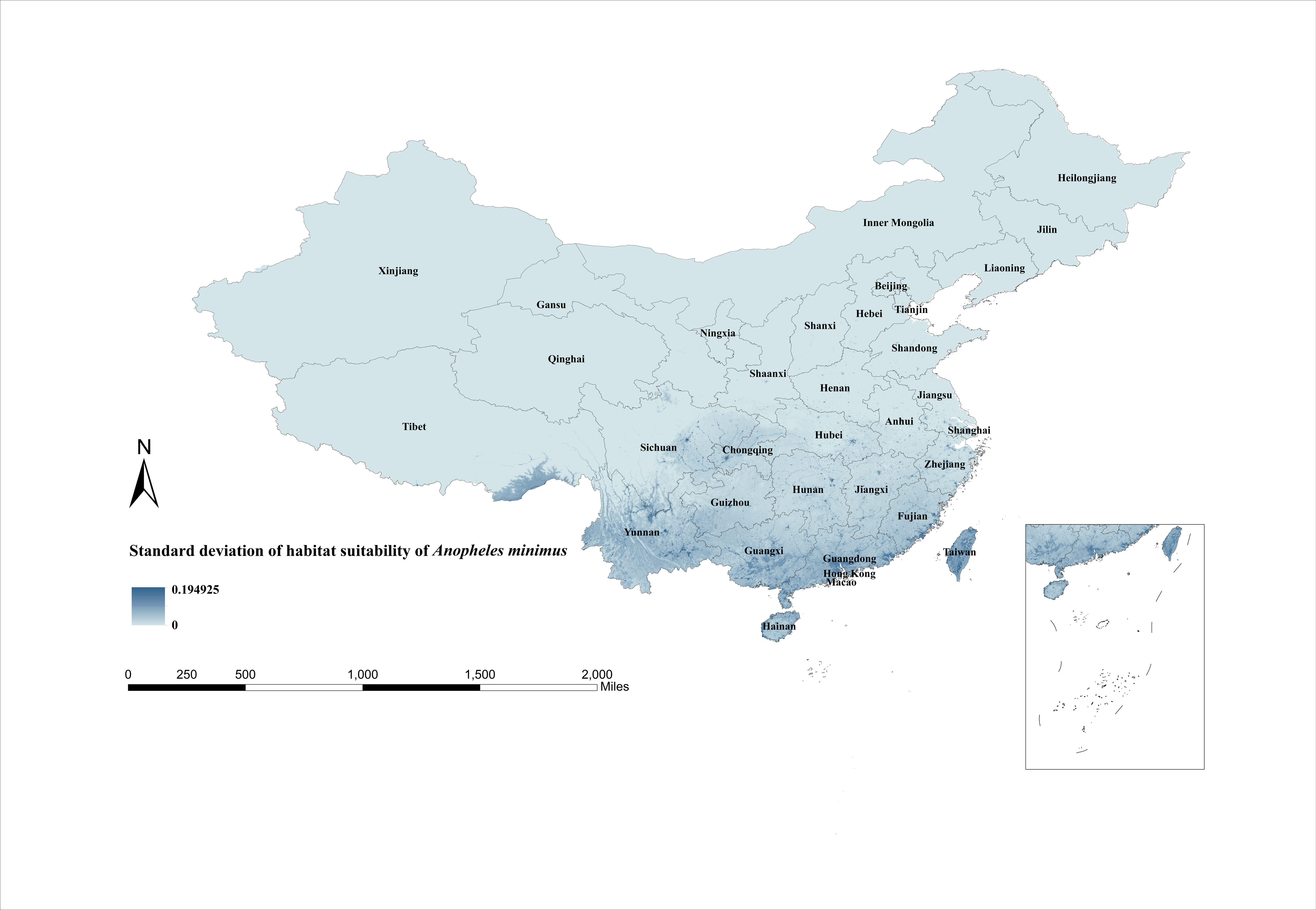

Supplement: S7 Fig — The map shows the standard deviation of output rasters of 10 repeated maxent runs. The darker colors depict areas of high standard deviation while lighter colors depict areas of low standard deviation. The base layer of the map is sourced from the National Catalogue Service For Geographic Information of the Ministry of Natural Resources of the People’s Republic of China (https://www.webmap.cn/mapDataAction.do?method=forw&resType=5&storeId=2&storeName=%E5%9B%BD%E5%AE%B6%E5%9F%BA%E7%A1%80%E5%9C%B0%E7%90%86%E4%BF%A1%E6%81%AF%E4%B8%AD%E5%BF%83&fileId=BA420C422A254198BAA5ABAB9CAAFBC1). (TIF) [file pntd.0011884.s007.tif]
